# Anterior cingulate cortex-related connectivity in first-episode schizophrenia: a spectral dynamic causal modeling study with functional magnetic resonance imaging

**DOI:** 10.3389/fnhum.2015.00589

**Published:** 2015-11-03

**Authors:** Long-Biao Cui, Jian Liu, Liu-Xian Wang, Chen Li, Yi-Bin Xi, Fan Guo, Hua-Ning Wang, Lin-Chuan Zhang, Wen-Ming Liu, Hong He, Ping Tian, Hong Yin, Hongbing Lu

**Affiliations:** ^1^Department of Radiology, Xijing Hospital, The Fourth Military Medical UniversityXi'an, China; ^2^Network Center, The Fourth Military Medical UniversityXi'an, China; ^3^Department of Psychiatry, Xijing Hospital, The Fourth Military Medical UniversityXi'an, China; ^4^School of Biomedical Engineering, The Fourth Military Medical UniversityXi'an, China

**Keywords:** schizophrenia, anterior cingulate cortex, functional magnetic resonance imaging, effective connectivity, spectral dynamic causal modeling

## Abstract

Understanding the neural basis of schizophrenia (SZ) is important for shedding light on the neurobiological mechanisms underlying this mental disorder. Structural and functional alterations in the anterior cingulate cortex (ACC), dorsolateral prefrontal cortex (DLPFC), hippocampus, and medial prefrontal cortex (MPFC) have been implicated in the neurobiology of SZ. However, the effective connectivity among them in SZ remains unclear. The current study investigated how neuronal pathways involving these regions were affected in first-episode SZ using functional magnetic resonance imaging (fMRI). Forty-nine patients with a first-episode of psychosis and diagnosis of SZ—according to the Diagnostic and Statistical Manual of Mental Disorders, Fourth Edition, Text Revision—were studied. Fifty healthy controls (HCs) were included for comparison. All subjects underwent resting state fMRI. We used spectral dynamic causal modeling (DCM) to estimate directed connections among the bilateral ACC, DLPFC, hippocampus, and MPFC. We characterized the differences using Bayesian parameter averaging (BPA) in addition to classical inference (*t*-test). In addition to common effective connectivity in these two groups, HCs displayed widespread significant connections predominantly involved in ACC not detected in SZ patients, but SZ showed few connections. Based on BPA results, SZ patients exhibited anterior cingulate cortico-prefrontal-hippocampal hyperconnectivity, as well as ACC-related and hippocampal-dorsolateral prefrontal-medial prefrontal hypoconnectivity. In summary, spectral DCM revealed the pattern of effective connectivity involving ACC in patients with first-episode SZ. This study provides a potential link between SZ and dysfunction of ACC, creating an ideal situation to associate mechanisms behind SZ with aberrant connectivity among these cognition and emotion-related regions.

## Introduction

Schizophrenia (SZ) affects approximately 1% of the population and is one of the leading causes of health burden all over the world (APA, 2013; Whiteford et al., 2013). It still remains unclear, however, regarding the pathogenesis of SZ, which has seriously hampered the efficacy of prevention and treatment for SZ. Understanding the neural basis of SZ is pivotal for shedding light on the neurobiological mechanisms behind this mental disease. The disconnection hypothesis suggests that the diverse symptoms of SZ are associated with abnormal neuronal connectivity between distinct regions (Friston and Frith, 1995; Friston, 1998; Stephan et al., 2009; Pettersson-Yeo et al., 2011). SZ, as a debilitating neuropsychiatric illness, involves both regional brain deficits and disruptions of communication among distinct brain regions (Friston and Frith, 1995; Stephan et al., 2009), including abnormal inter-hemispheric connectivity (Whitford et al., 2010; Chang et al., 2015). In the most recent years, increasing evidence has arisen supporting the notion that the structural and functional dysconnectivity within different brain regions is thought to account for the mechanism underlying SZ and its significant clinical and neuropathological heterogeneity on the basis of functional magnetic resonance imaging (fMRI) studies (Kubota et al., 2013; Voineskos et al., 2013; Liu et al., 2014a; Bastos-Leite et al., 2015; Genzel et al., 2015; Guo et al., 2015).

Structurally, on the one hand, SZ patients showed significant reduction of gray matter volume in the bilateral anterior cingulate cortex (ACC) as the largest effect size among all the areas investigated, as well as the bilateral posterior superior temporal gyri, bilateral inferior frontal gyri, left posterior amygdala-hippocampal complex (mostly hippocampus), and left insula (Yamasue et al., 2004). Concerning structural connectivity, major diffusion tensor imaging (DTI) findings highlighted a decreased fractional anisotropy (FA) value in the cingulate bundle, corpus callosum, and frontal and temporal white matter in chronic SZ (White et al., 2008; Pomarol-Clotet et al., 2010), whereas patients at the first-episode psychosis showed a decreased FA value in the inferior longitudinal fasciculus (Friedman et al., 2008) and a decreased mean diffusivity (MD) value in the left parahippocampal gyrus, left insula, and right ACC (Moriya et al., 2010). A previous meta-analysis of 15 DTI studies in SZ highlighted a significant FA reductions in two regions: the left frontal and temporal deep white matter (Ellison-Wright and Bullmore, 2009). Recently, Kubota et al. found SZ patients exhibited thalamo-orbitofrontal disconnection (Kubota et al., 2013). Specifically, reduced FA value in the right thalamo-orbitofrontal pathway and significantly positive correlation between FA value for this pathway and the right frontal polar and lateral orbitofrontal cortices were observed in this study. For patients with deficit SZ, they displayed disruption of white matter tracts at the right inferior longitudinal fasciculus, right arcuate fasciculus, and left uncinate fasciculus as compared with patients with nondeficit SZ (Voineskos et al., 2013). Furthermore, diffusion tensor tractography (DTT) analysis revealed a significant difference of connectivity between the bilateral medial prefrontal cortex (MPFC) and genu of the corpus callosum in SZ patients (Pomarol-Clotet et al., 2010).

Functionally, on the other hand, SZ is frequently characterized as not only a disorder of a large-scale brain connectivity but also a selective disruption of connectivity among central hub regions of the brain, but identifying its imaging-based connectomics is still challenging (Fornito et al., 2012; van den Heuvel et al., 2013). Resting state fMRI studies indicate widespread disconnectivity in the brain involved in the pathophysiology of SZ (Khadka et al., 2013; Mamah et al., 2013; Argyelan et al., 2014). For first-episode SZ patients, Bastos-Leite et al have reported reduced effective connectivity within the default mode network (DMN) using stochastic dynamic causal modeling (DCM), reflecting a reduced postsynaptic efficacy of prefrontal afferents (Bastos-Leite et al., 2015); Guo et al have demonstrated that patients revealed abnormal prefrontal-thalamic-cerebellar circuit using Granger causality analysis (GCA) (Guo et al., 2015). They found SZ may be associated with increased connectivity from the left MPFC or the right ACC to the sensorimotor regions and disrupted bilateral connections among sensorimotor regions, partly reflecting the effects of structural aberrancies in first-episode SZ on the prefrontal-thalamic-cerebellar circuit (Guo et al., 2015). Besides aberrant structural connectivity, the bilateral MPFC has been shown a marked failure of deactivation in SZ patients (Pomarol-Clotet et al., 2010). In addition, dorsolateral prefrontal cortex (DLPFC) is one of the most important cortical regions involved in the pathogenesis of SZ, and DLPFC-hippocampal formation dysconnectivity has been reported in SZ patients and links with the risk of developing SZ (Liu et al., 2014a). SZ patients also showed deficit in overnight memory consolidation associated with hippocampal-prefrontal connectivity (Genzel et al., 2015) and exhibited overactivation of DLPFC during social judgment (Mukherjee et al., 2014). In SZ, the connectivity between PFC and limbic regions (amygdala) was reduced during the resting state (Fan et al., 2013; Liu et al., 2014b), and absent and reversed or decreased during the processing of emotional stimuli (Das et al., 2007; Leitman et al., 2008). A reduced PFC-amygdala coupling was also associated with psychosis proneness in the general population (Modinos et al., 2010). Taken together, interactions among ACC, PFC (DLPFC and MPFC), and hippocampus have been crucially implicated in the neurobiology of SZ, and may represent a particular form of dysconnection in SZ. However, changes in connectivity patterns among these brain areas are largely unknown in SZ patients.

Effective connectivity is characterized by the causal (directed and weighted) influence of one brain region over another or itself. DCM, a technique used for measuring effective connectivity among different brain regions, is based on functional neuroimaging. Both functional and effective connectivity analyses are common methods used in resting state fMRI studies. However, DCM not only enables us to quantify the strength of connectivity among brain regions, but also allows the investigation of directed information flow from one region to another. An animal study has begun using spectral DCM to identify the pathophysiological theories of SZ recently (Moran et al., 2015). Here we used spectral DCM (Friston et al., 2014; Razi et al., 2015) to identify abnormal effective connectivity underlying SZ.

In the present study, we used spectral DCM to elucidate the effective connectivity among previously reported regions (bilateral ACC, DLPFC, hippocampi, and MPFC) associated with SZ, thereby providing a better understanding of the pathophysiological correlates of SZ. We hypothesized that directed connectivity involving these brain regions may be disturbed in SZ patients, predisposing to impairment of perceptual and cognitive functions and emotional behavior.

## Methods

### Subjects

The study sample consisted of 52 first-episode SZ patients from early intervention services within the outpatient clinic and inpatient department at Xijing Hospital, and 53 healthy controls (HCs) recruited by advertisement from the local community. Exclusion criteria comprised: pregnancy, major medical and neurological disorders, history of significant head trauma; illicit drug or alcohol abuse or dependence. Additional exclusion criteria for HCs included a current or past history of psychiatric illness and the presence of psychosis in first-degree relatives. The absence of any psychotic syndromes in HCs was confirmed using the Prodomal Questionnaire (Loewy et al., 2005). Two senior clinical psychiatrists performed the clinical-psychometric assessments—according to the Diagnostic and Statistical Manual of Mental Disorders, Fourth Edition, Text Revision (DSM-IV-TR)—with an interrater reliability >0.9. Patients were assessed with the Positive And Negative Syndrome Scale (PANSS) (Kay et al., 1987) on the day of scanning, as well as detailed information regarding past symptomatology acquired through patient interview and examination of patient's medical records. All participants gave written informed consent approved by the local Research Ethics Committee (Xijing Hospital, Fourth Military Medical University) after a complete description of this study.

### Magnetic resonance imaging acquisition

The fMRI images were acquired on a 3.0-T Siemens Magnetom Trio Tim scanner. During data acquisition, participants were asked to stay still in the scanner, keeping their eyes closed but not to fall asleep. The participants wore a custom-built MRI-compatible head coil fitted with foam pads and earplugs to minimize head motion and dampen scanner noise. Resting state functional scans were acquired with an echo planar imaging (EPI) sequence using the following parameters: repetition time = 2000 ms, echo time = 30 ms, filed of view = 220 mm × 220 mm, matrix = 64 × 64, flip angle = 90°, number of slices = 33, slice thickness = 4 mm, section gap = 0.6 mm. The whole scanning process lasted for 8 min and 240 scans were acquired for each subject.

### Data preprocessing

Images were preprocessed using SPM8 (http://www.fil.ion.ucl.ac.uk/spm/software/spm8/). For each subject, fMRI scans were first realigned to correct for head motion. Interscan motion was assessed with translation/rotation, and an exclusion criterion (>2.5 mm translation and/or >2.5° rotation in each direction) was set. Three SZ patients and three HCs met the criteria and were excluded from further analyses, resulting in that eventual 49 SZ patients and 50 HCs were included. Realigned images were then spatially normalized to the Montreal Neurological Institute space and finally smoothed using an 8 mm full width at half-maximum Gaussian kernel.

### General linear model and region of interest

At the first (within subject) level, a general linear model (GLM) was constructed for each participant. Fluctuations in neuronal activity will models with cosine basis functions. In addition, the six motion parameters from the realignment procedure and, one constant regressor modeling the baseline, and cosine basis functions were included in the GLM. See the resulting constant images used for constraining the ROI extraction step in the spectral DCM (Supplementary Figure 1).

**Figure 1:**
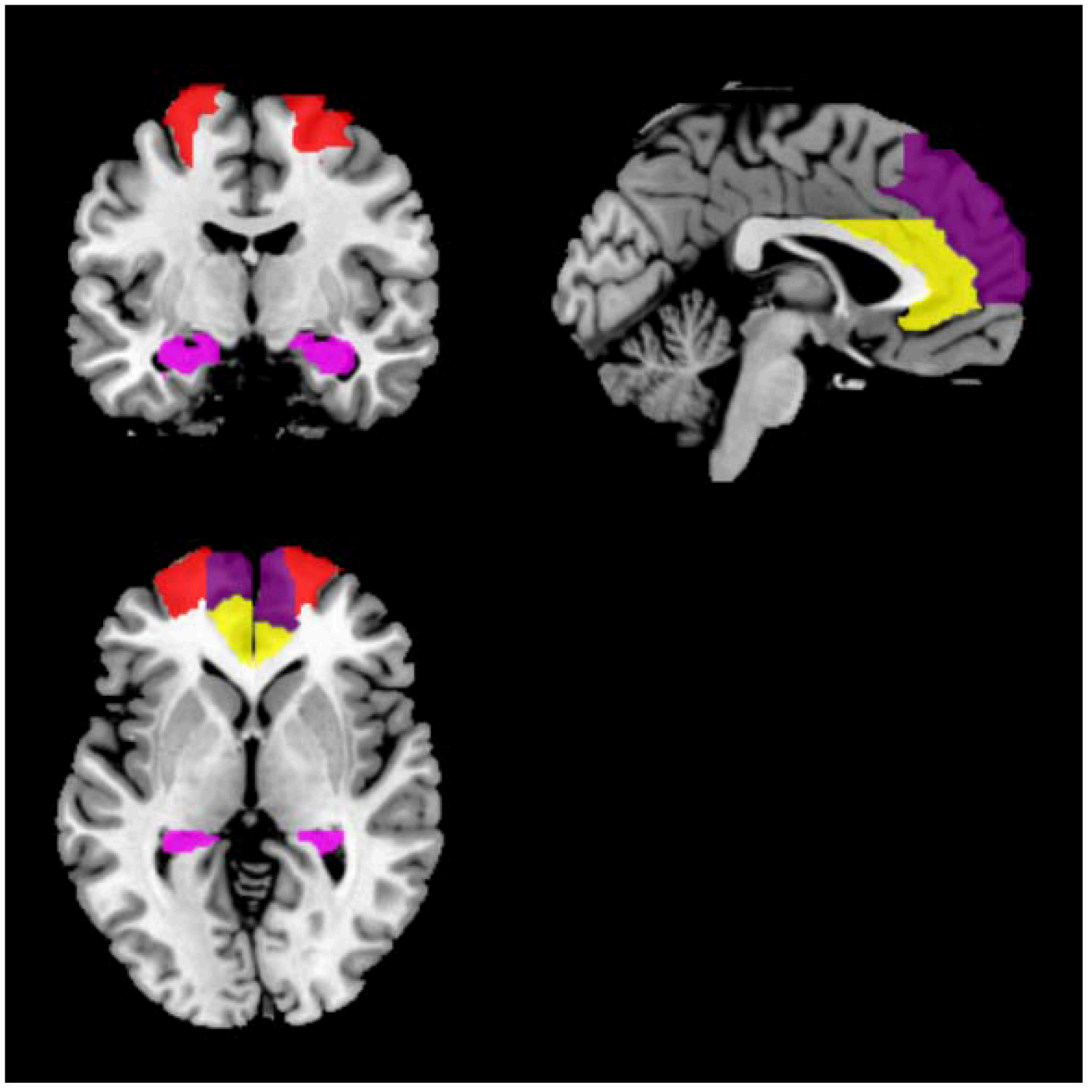
**Locations of the masks**. Red indicates DLPFC; yellow indicates ACC; dark purple indicates MPFC; violet indicates the hippocampus.

For each participant, symmetric eight regions of interest (ROIs) including bilateral ACC, DLPFC, hippocampi, and MPFC were selected. For each region, a mask was created using the WFU PickAtlas Tool and the automated anatomical labeling atlas template (Version 3.0.4, http://www.nitrc.org/projects/wfu_pickatlas/) (Tzourio-Mazoyer et al., 2002; Maldjian et al., 2003, 2004). For each ROI, subject-specific time series were extracted from a region defined by a thresholded SPM testing for the baseline and masked using the corresponding ROI from the WFU PickAtlas Tool (Figure 1). We used masks from the atlas for extraction of time series from the ROIs. All the voxels within the masks were used and first principle component was used as the extracted signal.

### Spectral dynamic causal modeling

Effective connectivity among the bilateral ACC, DLPFC, hippocampi, and MPFC was investigated using spectral DCM as described elsewhere (Friston et al., 2014). In the absence of a particular hypothesis or model space we used the fully connected model for an exploratory analysis of all possible reduced models, without one or more connections: after the full DCM for each participant was inverted, we employed a network discovery procedure using Bayesian model reduction (BMR) (Friston and Penny, 2011) to find the best model that explains the data. This procedure tests every possible model nested within the fully connected model. The model with the highest posterior probability is chosen as the winning model during this procedure. This BMR procedure is an efficient way to score a large model space without having to invert every reduced model. A fully connected model was constructed for each subject. This model was then inverted using generalized filtering (Friston et al., 2010). The model selection procedure was used to identify the model best explaining how the data are generated. Thus, we used a network discovery scheme in order to identify the optimal model pooling over all subjects (Friston et al., 2011). Model evidence of a fully connected model was used to approximate the model evidence of all the possible models and search for the model with the largest evidence. This network discovery-based model selection method can find the best model in the whole model space only by estimating parameters of a fully connected model (Li et al., 2012).

On the basis of spectral DCM analysis, the connection strength described the strength of a coupling according to the rate at which neuronal responses were induced in the target region (in other words connection strengths are effectively rate constants in 1/s, Hz). The resulting (maximum *a posteriori*) estimates of connectivity were then treated as summary statistics for classical random effects inference at the second (between subject) level using appropriate *t*-tests. We reported (Bonferroni corrected) *P*-values for all other connections to demonstrate the specificity of the differences. To see whether these differences could be estimated and detected reliably, we characterized the differences using Bayesian parameter averaging (BPA) in addition to classical inference (*t*-test) (Friston et al., 2014; Razi et al., 2015). Then, we used BPA for each group separately after network discovery procedure. See the flowchart of our each step (Supplementary Figure 2).

### Correlation analyses

To examine the correlations between effective connectivity and patients' symptomology scores, Pearson correlation coefficients were tentatively computed to test the relationship between connection strengths and PANSS positive, negative, general, and total scores in SZ patients.

## Results

### Clinical data

The demographic and clinical data are shown in Table 1. No significant difference was present between SZ patients and HCs on any demographic variables.

**Table 1:** **Demographic and clinical characteristics of first-episode SZ patients (***n*** = 49) and HCs (***n*** = 50)**.

**Characteristics**	**SZ Patients**	**HCs**	**Statistics**	***P***
Age	26 ± 6	27 ± 4	*t* = 1.57	0.12
Sex (male/female)	29/20	31/19	χ^2^ = 0.08	0.77
Ethnicity	Han (Chinese)	Han (Chinese)		
Handedness (right/left)	49/0	50/0		
Duration of illness (months)	10 ± 14	—		
PANSS total score	162 ± 27	—		
PANSS positive score	24 ± 8	—		
PANSS negative score	24 ± 7	—		
PANSS general psychopathology	49 ± 9	—		

*PANSS, Positive and Negative Syndrome Scale*.

### Network discovery-based model selection findings

**Figure 2:**
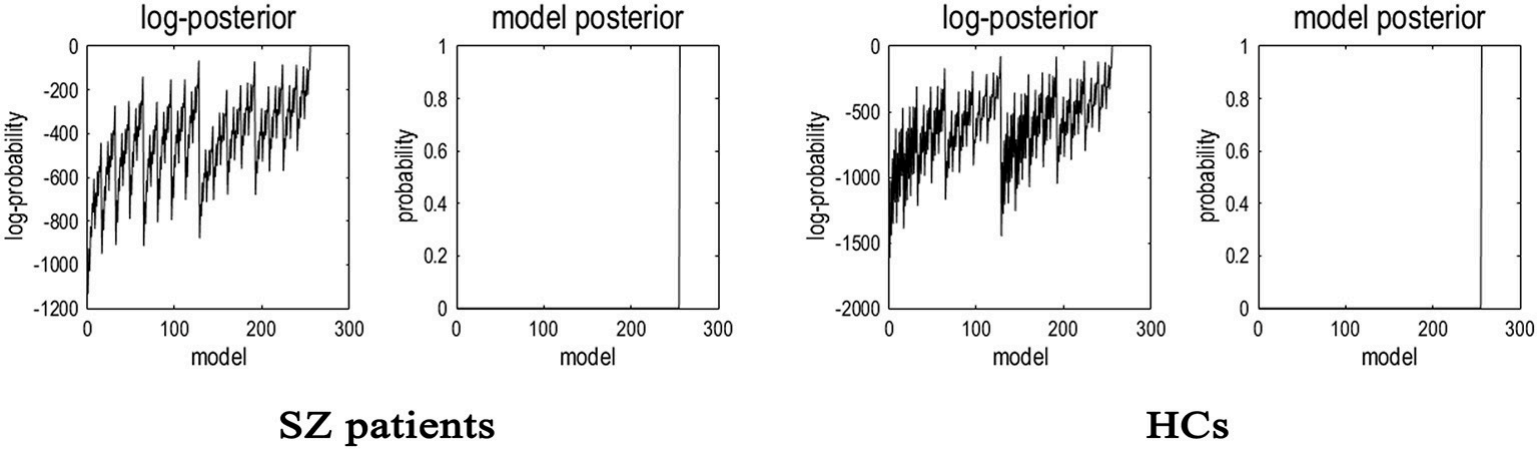
**Results of the ***post-hoc*** optimization or network findings**. The corresponding conditional parameter estimates were shown over the 49 (intrinsic and extrinsic) connections. The profiles of model evidences are shown with the posterior probability for each model. In both groups, the full model had a probability of almost 1 and a log-probability of almost 0. As shown in the figure, the model with the highest evidence was the fully connected model based on the results of *post-hoc* optimization or network findings.

Having inverted a fully connected model with full extrinsic connectivity, the log model evidence for all reduced models (models with one or more missing connections) was then assessed. Figure 2 shows the network discovery procedure compared the evidence of all reduced models for each group and the results of *post-hoc* optimization. The left panel is for SZ patients and right panel refers to HCs. The fully connected model was the full model with the highest evidence. The procedure selected the fully connected model as the best model with a posterior probability of almost 1. The fully connected model had 49 parameters describing the extrinsic connections between nodes and the intrinsic (self-connections) within nodes. This suggested that the fully connected model was the best explanation for these data, indicating eligible and rational ROI selection.

### Effective connectivity

**Figure 3:**
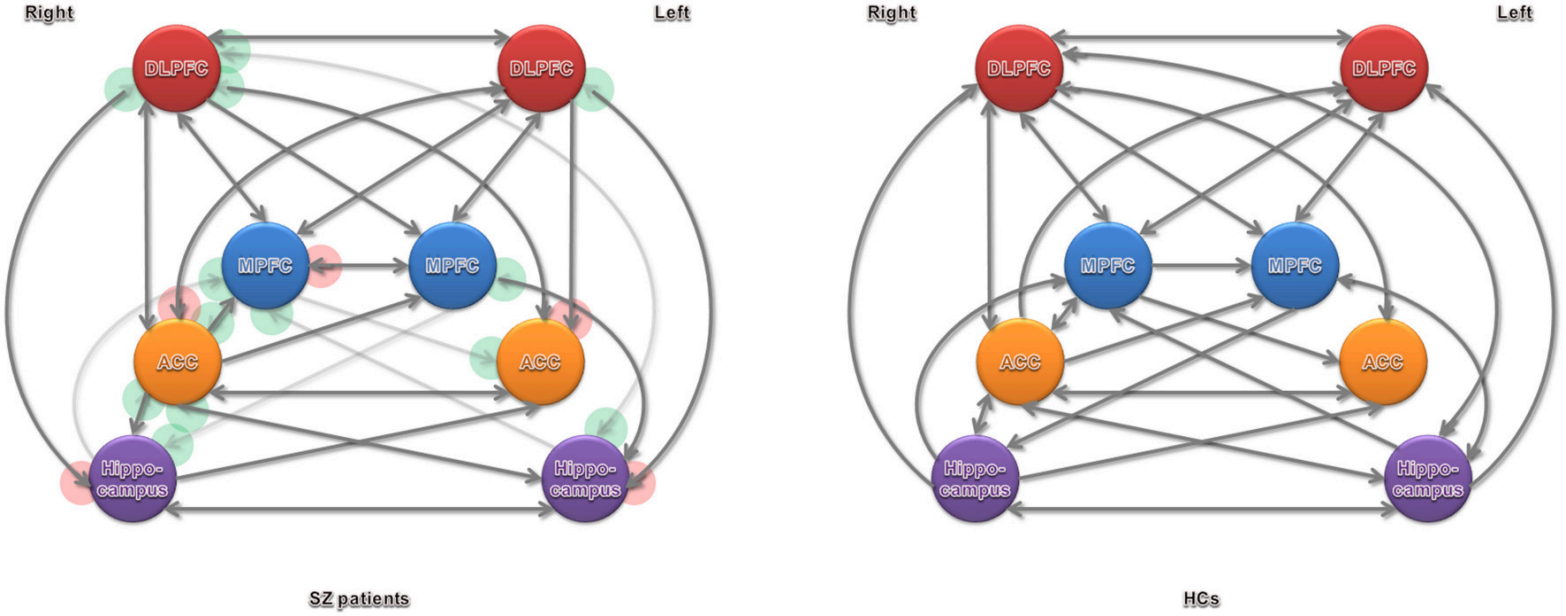
**Significant effective connectivity (at the group level) among ROIs in the SZ patients and HCs**. Arrowheads in the red circles refers to significant connections detected in SZ patients but not in HCs, and green in HCs but not in SZ patients.

Significant connections at the group level (one-sample *t*-test at *P*-value of 0.05, Bonferroni corrected for multiple comparisons) are shown in Figure 3 and Table 2 (in terms of simple main effects within group). See the *t*- and *P*-values for strength of connections at the group level (Supplementary Table 1). However, two-sample *t*-test did not show significant difference between SZ patients and HCs (*P* > 0.05, Bonferroni corrected for multiple comparisons, i.e., 0.05/56).

**Table 2:** **Strength of connections in first-episode SZ patients and HCs**.

**Connections**	**First-episode SZpatients**	**HCs**	***t*^#^**	***P*^#^**
Left ACC-right ACC	0.1145±0.2783^*^	0.1005±0.2236^*^	0.28	0.78
Left ACC-left DLPFC	−0.0162±0.1936	−0.0179±0.2197	0.04	0.97
Left ACC-right DLPFC	−0.0453±0.2523	−0.0861±0.2426^*^	0.82	0.41
Left ACC-left MPFC	−0.0261±0.2156	0.0395±0.2169	−1.51	0.13
Left ACC-right MPFC	−0.0346±0.2004	−0.0494±0.2745	0.31	0.76
Left ACC-left hippocampus	−0.0367±0.2116	−0.0223±0.2075	−0.34	0.73
Left ACC-right hippocampus	0.0047±0.3136	−0.0253±0.2053	0.56	0.57
Right ACC-left ACC	0.4739±0.2108^*^	0.4824±0.1969^*^	−0.21	0.84
Right ACC-left DLPFC	0.1541±0.1620^*^	0.1581±0.1650^*^	−1.12	0.90
Right ACC-right DLPFC	0.1977±0.1815^*^	0.1597±0.1472^*^	1.15	0.25
Right ACC-left MPFC	0.1866±0.1749^*^	0.1981±0.1754^*^	−0.33	0.74
Right ACC-right MPFC	0.2116±0.1485^*^	0.2222±0.1694^*^	−0.33	0.74
Right ACC-left hippocampus	0.0862±0.1655^*^	0.0494±0.1520^*^	1.15	0.25
Right ACC-right hippocampus	0.1189±0.2312^*^	0.0509±0.1349^*^	1.79	0.08
Left DLPFC-left ACC	0.0929±0.1856^*^	0.0577±0.2022	0.90	0.37
Left DLPFC-right ACC	0.0943±0.2102^*^	0.0482±0.2590	0.97	0.33
Left DLPFC-right DLPFC	0.1042±0.1694^*^	0.1560±0.1749^*^	−1.50	0.14
Left DLPFC-left MPFC	0.2195±0.1778^*^	0.2554±0.1708^*^	−1.02	0.31
Left DLPFC-right MPFC	0.1365±0.1693^*^	0.1572±0.1682^*^	−0.61	0.54
Left DLPFC-left hippocampus	0.0759±0.1713^*^	0.0028±0.1299	2.40	0.02
Left DLPFC-right hippocampus	0.0449±0.1617	0.0206±0.1304	0.82	0.41
Right DLPFC-left ACC	0.0724±0.2217^*^	0.1254±0.2171^*^	−1.20	0.23
Right DLPFC-right ACC	0.0946±0.2593^*^	0.1569±0.3082^*^	−1.09	0.28
Right DLPFC-left DLPFC	0.1538±0.2354^*^	0.1953±0.2196^*^	−0.91	0.37
Right DLPFC-left MPFC	0.1323±0.2054^*^	0.1564±0.2132^*^	−0.57	0.57
Right DLPFC-right MPFC	0.2201±0.2080^*^	0.2981±0.2186^*^	−1.82	0.07
Right DLPFC-left hippocampus	0.0531±0.1870	0.0545±0.1743^*^	−0.04	0.97
Right DLPFC-right hippocampus	0.0535±0.1575^*^	0.0332±0.1783	0.60	0.55
Left MPFC-left ACC	0.0455±0.2294	−0.0221±0.2118	1.52	0.13
Left MPFC-right ACC	0.0257±0.2497	−0.0511±0.2471	1.54	0.13
Left MPFC-left DLPFC	0.1033±0.1810^*^	0.1012±0.1987^*^	0.05	0.96
Left MPFC-right DLPFC	−0.0040±0.1963	−0.0318±0.2056	0.69	0.49
Left MPFC-right MPFC	0.0687±0.2102^*^	0.0385±0.2139	0.71	0.48
Left MPFC-left hippocampus	0.0611±0.1920^*^	−0.0598±0.1663^*^	3.35	0.00
Left MPFC-right hippocampus	−0.0301±0.2014	−0.0514±0.1499^*^	0.60	0.55
Right MPFC-left ACC	0.0130±0.2290	0.0861±0.2633^*^	−1.47	0.14
Right MPFC-right ACC	0.0613±0.2475	0.1037±0.2857^*^	−0.79	0.43
Right MPFC-left DLPFC	0.1146±0.1663^*^	0.1136±0.1946^*^	0.03	0.98
Right MPFC-right DLPFC	0.1751±0.1557^*^	0.1965±0.1744^*^	−0.64	0.52
Right MPFC-left MPFC	0.1503±0.1764^*^	0.1969±0.2051^*^	−1.21	0.23
Right MPFC-left hippocampus	0.0425±0.1591	−0.0099±0.1367	1.76	0.08
Right MPFC-right hippocampus	0.0217±0.1825	−0.0071±0.1460	0.87	0.39
Left hippocampus-left ACC	0.0553±0.2922	0.0695±0.2859	−0.24	0.81
Left hippocampus-right ACC	0.0986±0.3714	0.1038±0.3491^*^	−0.07	0.94
Left hippocampus-left DLPFC	0.0305±0.2759	0.1099±0.3088^*^	−1.35	0.18
Left hippocampus-right DLPFC	0.0393±0.2713	0.1012±0.3161^*^	−1.05	0.30
Left hippocampus-left MPFC	0.0620±0.2660	0.0825±0.2795^*^	−0.37	0.71
Left hippocampus-right MPFC	0.0300±0.2834	0.0961±0.3044^*^	−1.12	0.27
Left hippocampus-right hippocampus	0.1672±0.3269^*^	0.2188±0.3074^*^	−0.81	0.42
Right hippocampus-left ACC	0.0934±0.3193^*^	0.0823±0.2694^*^	0.19	0.85
Right hippocampus-right ACC	0.0923±0.3510	0.1485±0.3468^*^	−0.80	0.43
Right hippocampus-left DLPFC	0.0461±0.3041	0.0844±0.3066	−0.62	0.53
Right hippocampus-right DLPFC	0.0693±0.2612	0.1085±0.3097^*^	−0.68	0.50
Right hippocampus-left MPFC	0.0735±0.2661	0.0675±0.2667	0.11	0.91
Right hippocampus-right MPFC	0.0457±0.2851	0.0951±0.2867^*^	−0.86	0.39
Right hippocampus-left hippocampus	0.2198±0.2791^*^	0.2513±0.2341^*^	−0.61	0.54

* *Significant effective connectivity at the group level (P < 0.05, Bonferroni corrected)*.

# *Between the group level. ACC, anterior cingulate cortex; DLPFC, dorsolateral prefrontal cortex; MPFC, medial prefrontal cortex*.

**Figure 4:**
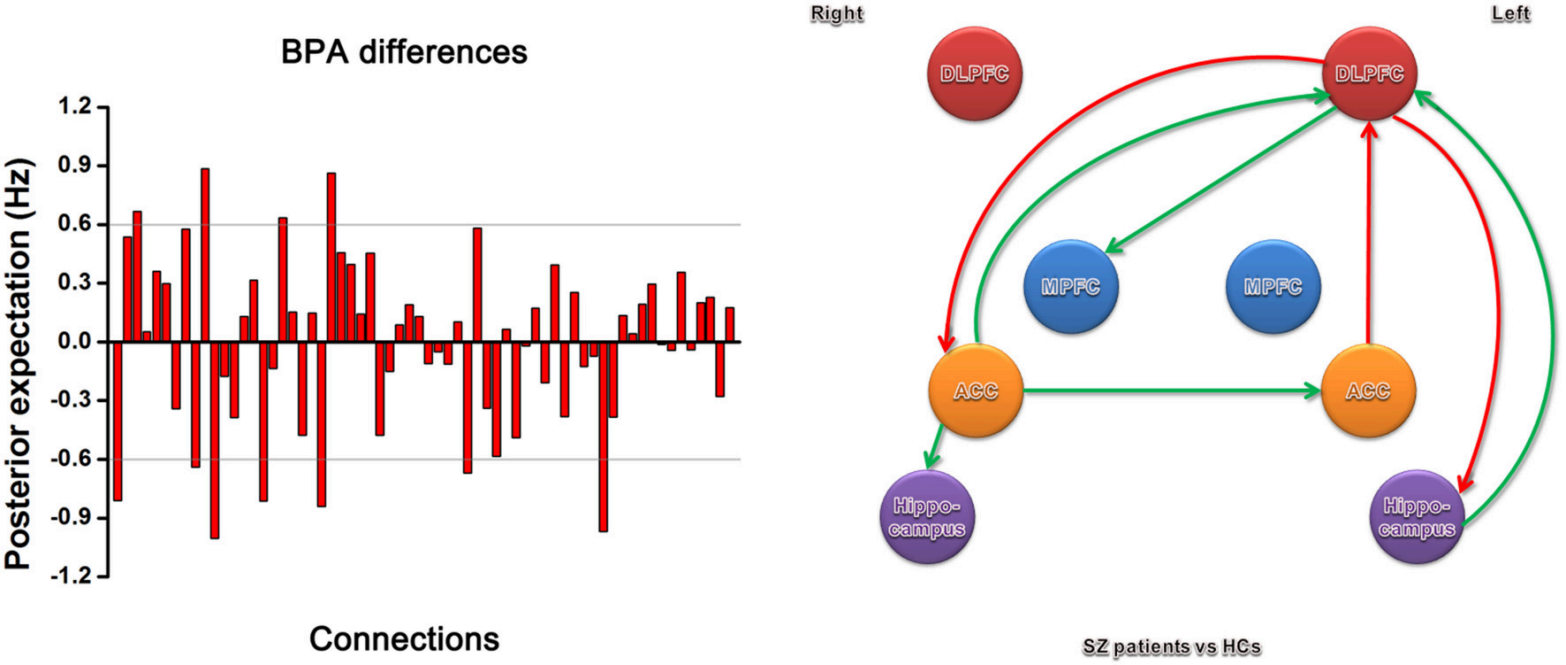
**Significant effective connectivity (between the group level) among ROIs in the SZ patients and HCs**. The left panel shows the BPA of the differences for spectral DCM. The right panel shows only those edges on the graph that survive the threshold of 0.6 Hz in the left panel, i.e., the increased (red) and decreased (green) connections in SZ patients relative to HCs.

The BPA results of the effective connectivity are shown in Figure 4. We set the threshold to 0.6 Hz. SZ patients exerted increased connections from the left ACC to left DLPFC, and from the left DLPFC to right ACC and left hippocampus, but decreased connections from the right ACC to left ACC, left DLPFC, and right hippocampus, from the left hippocampus to left DLPFC, and from the left DLPFC to right MPFC.

### Correlation analyses

Finally, we calculated the correlation between patients' symptomology scores and the strength of all the connections in SZ patients. But there was no significant correlation between PANSS score and strength of connections with differences in SZ patients relative to HCs (*P* > 0.05). On the basis of the current findings, these results do not provide a symptom-based validation of the quantitative effective connectivity estimates involving ACC, PFC, and hippocampus; in that they do not demonstrate the effective connectivity estimates among brain regions investigated in our present study have predictive validity in relation to clinical phenotype.

## Discussion

To our knowledge, this is the first study to demonstrate the effective connectivity among ACC, PFC, and hippocampus in patients with first-episode SZ using spectral DCM. In SZ patients, excessive effective connectivity is seen from the left ACC to left DLPFC, and from the left DLPFC to left hippocampus and right ACC; deficit effective connectivity is detected from the right ACC to left ACC, left DLPFC, and right hippocampus, as well as from the left hippocampus to left DLPFC and from the left DLPFC to right MPFC. Our results indicate abnormal effective connectivity involving ACC in first-episode SZ patients.

In the past decade, many studies have ever focused on structural and functional alterations of ACC, PFC, and hippocampus in SZ, and abnormalities of these regions in patients with SZ have been repeatedly reported. Compared with HCs, SZ patients showed significant gray matter volume reduction in ACC and hippocampus (Yamasue et al., 2004; Benedetti et al., 2011). A study combining fMRI and DTI demonstrated altered prefrontal structure-function relationships in SZ (Schlösser et al., 2007). It highlights a potential relationship between anatomical changes in a frontal-temporal anatomical circuit and functional alterations in the PFC. Brain fMRI neural responses to a face-matching paradigm and regional gray matter volumes were studied in the amygdala, hippocampus, ACC, and PFC (Benedetti et al., 2011). As compared with HCs, patients with chronic undifferentiated SZ reported higher adverse childhood experiences, proportionally leading to decreasing responses in the amygdala and hippocampus, and increasing responses in PFC and ACC. Lui et al found a decreased amplitude of low-frequency fluctuation in ACC and reduced functional connectivity between the left ACC and right middle temporal gyrus using resting state fMRI (Lui et al., 2015). Altered functional connections associated with ACC, MPFC, hippocampus, thalamus, and cerebellum were also observed in SZ patients (Yu et al., 2013). Both at resting state and during emotional stimuli, abnormalities can be observed in PFC-amygdala connection (Das et al., 2007; Leitman et al., 2008; Fan et al., 2013; Liu et al., 2014b). PFC can be subdivided into MPFC and DLPFC. SZ patients revealed abnormal activation in the bilateral MPFC (Pomarol-Clotet et al., 2010). With the exception of MPFC, SZ patients also exhibited overactivation of DLPFC during social judgment (Mukherjee et al., 2014). During face-matching paradigms, aberrancies were detected in DLPFC-amygdala connection using DCM (Diwadkar et al., 2012; Vai et al., 2015). These findings indicate the importance of ACC, PFC, and hippocampal abnormalities in the pathophysiology of SZ.

From the perspective of neurochemical abnormalities of neurotransmitters or their receptors, regions mentioned above are in line with studies determining *in vivo* glutamate and glutamine concentrations in SZ patients' brains. Glutamatergic dysfunction has been implicated in the pathophysiology of SZ. Chun et al. identified that an SZ-associated microdeletion disrupted glutamatergic synaptic transmission at thalamocortical projections to the auditory cortex in SZ mouse models (Chun et al., 2014). Likewise, a previous study reported increased levels of glutamate in prefrontal and hippocampal areas in patients with SZ using magnetic resonance spectroscopy (van Elst et al., 2005). Magnetic resonance spectroscopy and tissue protein concentrations sampling SZ patients *in vivo* and postmortem brain tissue *in vitro*, respectively, together suggest lower glutamate level in dentate gyrus, implicating the excitatory system within hippocampus in the pathophysiology of SZ (Stan et al., 2015). With the exception of altered concentration of glutamate, in DLPFC, decoupling of the sum of glutamate and glutamine and N-acetylaspartate was observed in SZ patients (Coughlin et al., 2015). These findings provide strong evidence supporting the hypothesis of glutamatergic dysfunction within PFC and hippocampus in SZ.

From functional aspect, SZ is a mental illness involved in abnormality of emotional responses and difficulty with social interactions. Both resting-state functional connectivity analysis by Mamah et al. (2013) and effective connectivity analyses during face-matching paradigms by Diwadkar et al. (2012) and Vai et al. (2015) indicate dysfunction in the connections between networks involved in cognitive and emotional processing in the pathophysiology SZ. ACC might interact with other cortical and subcortical structures as a part of the circuits involved in the regulation of mental and emotional activity. Hoptman et al. investigate the construct of urgency in association with aggression in individuals with SZ or schizoaffective disorder and its underlying neural circuitry. Their findings revealed that greater urgency was related to lower cortical thickness and functional connectivity within the medial/lateral orbitofrontal and inferior frontal regions, and rostral ACC (Hoptman et al., 2014). Clinically, patients with chronic SZ often show lack of motivation and difficulty with decision-making. At the neural level, the orbitofrontal cortex and ACC are thought to interact, together, to form a network involved in emotional processing and mediating emotion and social behavior (Ohtani et al., 2014). Reductions in FA were observed in connections between the left anterior medial orbitofrontal cortex and rostral ACC, and between bilateral posterior medial orbitofrontal cortex and rostral ACC in SZ patients relative to HCs. In addition, reduced FA was correlated with more severe anhedonia-asociality and avolition-apathy using the Scale for the Assessment of Negative Symptoms, which suggests that ACC may be pivotal in understanding aberrant emotional responses and social behavior in SZ patients. Finally, cognitive deficits are a defining feature of SZ, affecting quality of life and functional outcomes in work, relationships, and independent living. While viewing faces, SZ patients showed significantly weaker deactivation of MPFC, including ACC, and decreased activation in the left cerebellum, compared to controls (Mothersill et al., 2014). Considering the role of ACC in processing negative emotion, weaker deactivation of this region in SZ patients while viewing faces may lead to an elevated perception of social threat. Future studies examining the neurobiology of cognitive function in SZ using fMRI may aid in establishing strategy of targeted treatment.

Additionally, previous studies consistently demonstrated the key role of the dorsal ACC and DLPFC in cognitive control (Carter and van Veen, 2007). For these regions aberrant activation patterns in association with deficient behavioral performance were observed in SZ (Minzenberg et al., 2009). Our effective connectivity study showed decreased bilateral connectivity between ACC and DLPFC in first-episode SZ patients. Through effective connectivity and white matter connectivity analysis, combined utilization of DCM and diffusion tensor imaging provides some support for that weaker connectivity involved in ACC may be the neural basis of specific cognitive impairments in SZ (Wagner et al., 2015). Cognitive deficits are considered as a core feature of SZ (Elvevåg and Goldberg, 2000). Converging evidence from fMRI studies may shed light on that ACC and DLPFC play a crucial role in cognitive function in SZ, albeit the neural underpinnings of impaired cognition in SZ remain uncertain.

In addition, as stated previously, spectal DCM has started to be used in a recent animal studies to disclose the pathophysiology of SZ. Administration of ketamine disrupted desynchronized electrical activity between MPFC and hippocampus (Moran et al., 2015). Our current study also demonstrated hippocampal-prefrontal hypoconnectivity *in vivo* in first-episode SZ. Strictly speaking, SZ patients showed decreased hippocampal-dorsolateral prefrontal-medial prefrontal connectivity. To some extent, connections associated with PFC and hippocampus that we have found, show promise as an intermediate link in this neural pathway for SZ.

In this study, we found no significant correlation between these connections with differences and PANSS scores. The lack of significant correlation may relate to our modest sample size of SZ patients.

The strengthen of our study is that there may be no potential confounds related to medications and state of illness due to first-episode SZ patients. The inclusion criterion minimized the influences of medication, cohort effects and illness-related environmental factors. Nevertheless, we acknowledge that there were several limitations. First, significance in one group and not in the other group does not imply that there are differences between these two groups. Group difference was not significant in our study, which was the major limitation. Second, we enrolled a large sample size of participants in our study, thus doubling or tripling the numbers investigated in most previous fMRI studies. But larger sample and multi-center studies, like some recent investigations (Ivleva et al., 2013; Skudlarski et al., 2013; Meda et al., 2014; Bois et al., 2015), are desirable to confirm our present findings. We hope to extend this research to a larger patient population, which will increase statistical efficiency and sensitivity to more subtle changes. Third, effective connectivity was only measured during the resting state without giving any tasks. A comparison study at resting state and active state might highlight the specificity of functional brain changes. Fourth, the current findings were only based on the changes of BOLD signal in SZ patients. In addition to BOLD-fMRI, a combination of multimodalities, including diffusion tensor imaging, magnetic resonance spectroscopy, electroencephalography, and positron emission tomography, might further strengthen the conclusion.

The present study characterized the abnormal ACC-related connectivity *in vivo* in first-episode SZ by means of spectral DCM, revealing anterior cingulate cortico-prefrontal-hippocampal hyperconnectivity, as well as ACC-related and hippocampal-dorsolateral prefrontal-medial prefrontal hypoconnectivity. Spectral DCM revealed abnormal effective connectivity involving ACC in patients with first-episode SZ. This suggests the SZ subjects fail to recruit these neural pathways. This study further provides a link between SZ and dysconnection hypothesis, creating an ideal situation to associate mechanisms behind SZ with aberrant connectivity among these cognition and emotion-related regions.

### Conflict of interest statement

The authors declare that the research was conducted in the absence of any commercial or financial relationships that could be construed as a potential conflict of interest.

## Supplementary material

Supplementary Figure 1 **Presentative images of fMRI first-level results**. The left is for one healthy control and the right is for one SZ patient.

Supplementary Figure 2 **Steps for data analysis**.
