# Supplementary figures and images for: Anterior cingulate cortex-related connectivity in first-episode schizophrenia: a spectral dynamic causal modeling study with functional magnetic resonance imaging
Source: Front Hum Neurosci. 2015 Nov 3;9:589. doi: 10.3389/fnhum.2015.00589 (PMC4630283; doi:10.3389/fnhum.2015.00589)

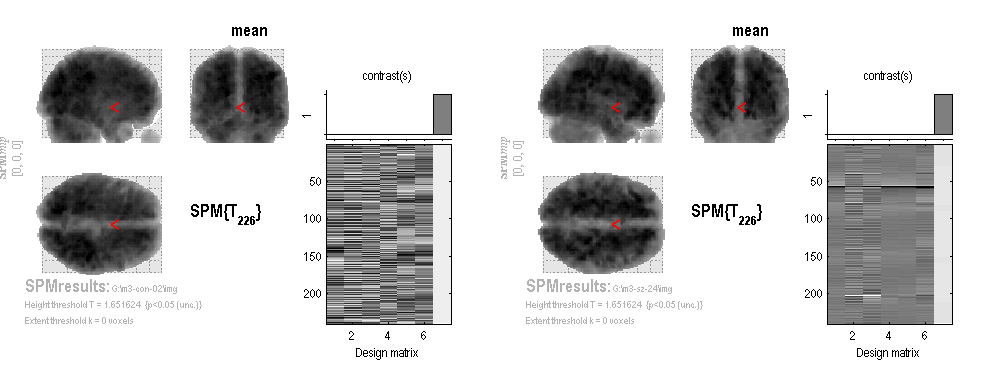

Supplement: Supplementary Figure 1 — Presentative images of fMRI first-level results. The left is for one healthy control and the right is for one SZ patient. [file Image1.TIF]

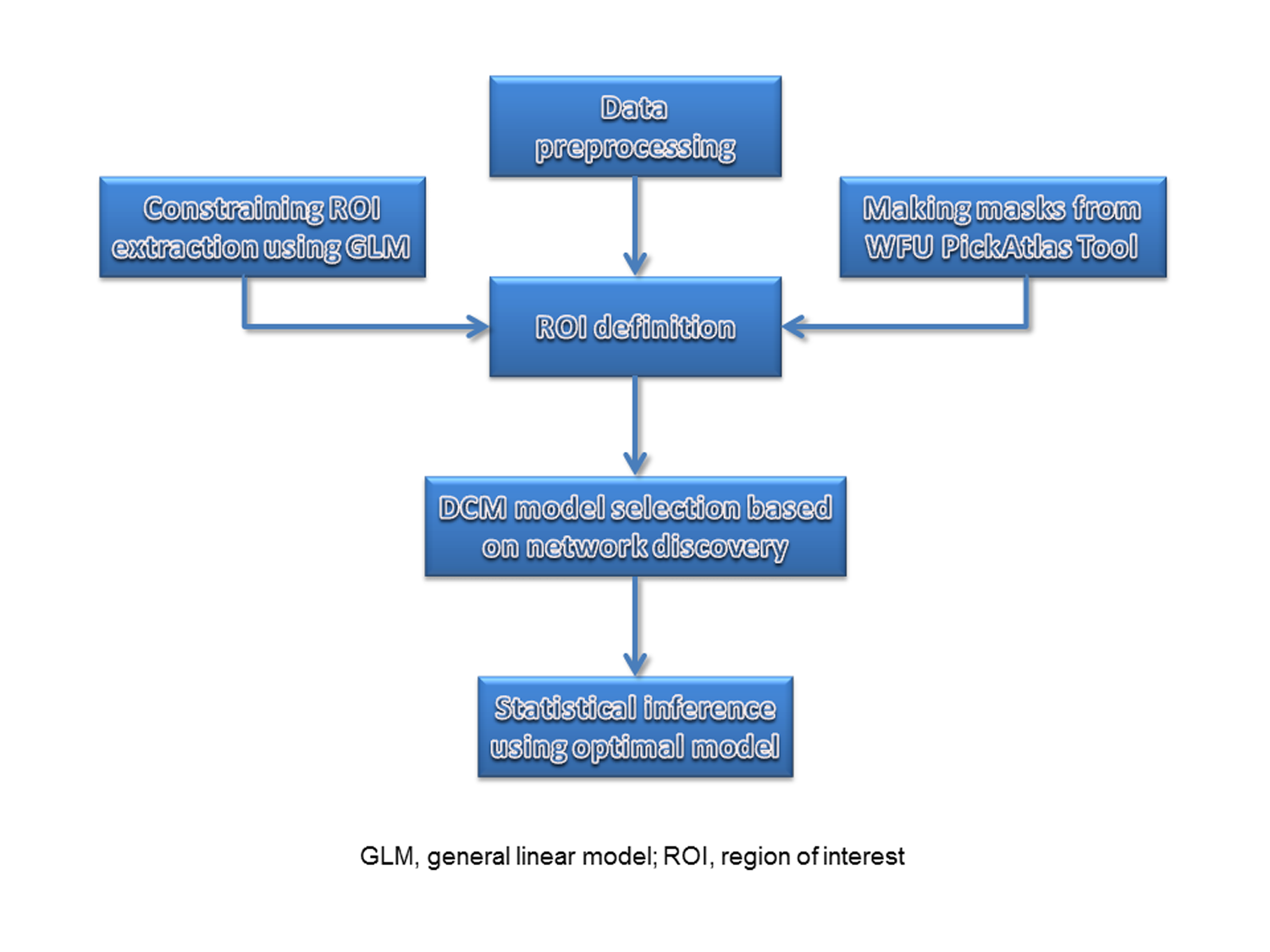

Supplement: Supplementary Figure 2 — Steps for data analysis. [file Image2.TIF]
