# Supplementary material for: Anterior cingulate cortex-related connectivity in first-episode schizophrenia: a spectral dynamic causal modeling study with functional magnetic resonance imaging
Source: Front Hum Neurosci. 2015 Nov 3;9:589. doi: 10.3389/fnhum.2015.00589 (PMC4630283; doi:10.3389/fnhum.2015.00589)
Supplement: Supplementary file 3 [file Table1.DOCX]

**Supplementary Table 1 | Strength of connections and t and P values at the group level in first-episode SZ patients and HCs.**

| **Connections** | **First-episode SZ Patients** | | |  | **HCs** | | |
| --- | --- | --- | --- | --- | --- | --- | --- |
|  | **Strength** | ***t*** | ***P*** |  | **Strength** | ***t*** | ***P*** |
| Left ACC-right ACC | 0.1145 ± 0.2783^*^ | 2.88 | 0.01 |  | 0.1005 ± 0.2236^*^ | 3.18 | 0.00 |
| Left ACC-left DLPFC | −0.0162 ± 0.1936 | −0.58 | 0.56 |  | −0.0179 ± 0.2197 | −0.58 | 0.57 |
| Left ACC-right DLPFC | −0.0453 ± 0.2523 | −1.26 | 0.21 |  | −0.0861 ± 0.2426^*^ | −2.51 | 0.02 |
| Left ACC-left MPFC | −0.0261 ± 0.2156 | −0.85 | 0.40 |  | 0.0395 ± 0.2169 | 1.29 | 0.20 |
| Left ACC-right MPFC | −0.0346 ± 0.2004 | −1.21 | 0.23 |  | −0.0494 ± 0.2745 | −1.27 | 0.21 |
| Left ACC-left hippocampus | −0.0367 ± 0.2116 | −1.22 | 0.23 |  | −0.0223 ± 0.2075 | −0.76 | 0.45 |
| Left ACC-right hippocampus | 0.0047 ± 0.3136 | 0.10 | 0.92 |  | −0.0253 ± 0.2053 | −0.87 | 0.39 |
| Right ACC-left ACC | 0.4739 ± 0.2108^*^ | 15.74 | 0.00 |  | 0.4824 ± 0.1969^*^ | 17.32 | 0.00 |
| Right ACC-left DLPFC | 0.1541 ± 0.1620^*^ | 6.66 | 0.00 |  | 0.1581 ± 0.1650^*^ | 6.77 | 0.00 |
| Right ACC-right DLPFC | 0.1977 ± 0.1815^*^ | 7.63 | 0.00 |  | 0.1597 ± 0.1472^*^ | 7.67 | 0.00 |
| Right ACC-left MPFC | 0.1866 ± 0.1749^*^ | 7.47 | 0.00 |  | 0.1981 ± 0.1754^*^ | 7.99 | 0.00 |
| Right ACC-right MPFC | 0.2116 ± 0.1485^*^ | 9.97 | 0.00 |  | 0.2222 ± 0.1694^*^ | 9.27 | 0.00 |
| Right ACC-left hippocampus | 0.0862 ± 0.1655^*^ | 3.65 | 0.00 |  | 0.0494 ± 0.1520^*^ | 2.30 | 0.03 |
| Right ACC-right hippocampus | 0.1189 ± 0.2312^*^ | 3.60 | 0.00 |  | 0.0509 ± 0.1349^*^ | 2.67 | 0.01 |
| Left DLPFC-left ACC | 0.0929 ± 0.1856^*^ | 3.50 | 0.00 |  | 0.0577 ± 0.2022 | 2.02 | 0.05 |
| Left DLPFC-right ACC | 0.0943 ± 0.2102^*^ | 3.14 | 0.00 |  | 0.0482 ± 0.2590 | 1.32 | 0.19 |
| Left DLPFC-right DLPFC | 0.1042 ± 0.1694^*^ | 4.31 | 0.00 |  | 0.1560 ± 0.1749^*^ | 6.31 | 0.00 |
| Left DLPFC-left MPFC | 0.2195 ± 0.1778^*^ | 8.64 | 0.00 |  | 0.2554 ± 0.1708^*^ | 10.58 | 0.00 |
| Left DLPFC-right MPFC | 0.1365 ± 0.1693^*^ | 5.64 | 0.00 |  | 0.1572 ± 0.1682^*^ | 6.61 | 0.00 |
| Left DLPFC-left hippocampus | 0.0759 ± 0.1713^*^ | 3.10 | 0.00 |  | 0.0028 ± 0.1299 | 0.15 | 0.88 |
| Left DLPFC-right hippocampus | 0.0449 ± 0.1617 | 1.94 | 0.06 |  | 0.0206 ± 0.1304 | 1.12 | 0.27 |
| Right DLPFC-left ACC | 0.0724 ± 0.2217^*^ | 2.29 | 0.03 |  | 0.1254 ± 0.2171^*^ | 4.08 | 0.00 |
| Right DLPFC-right ACC | 0.0946 ± 0.2593^*^ | 2.55 | 0.01 |  | 0.1569 ± 0.3082^*^ | 3.60 | 0.00 |
| Right DLPFC-left DLPFC | 0.1538 ± 0.2354^*^ | 4.57 | 0.00 |  | 0.1953 ± 0.2196^*^ | 6.29 | 0.00 |
| Right DLPFC-left MPFC | 0.1323 ± 0.2054^*^ | 4.51 | 0.00 |  | 0.1564 ± 0.2132^*^ | 5.19 | 0.00 |
| Right DLPFC-right MPFC | 0.2201 ± 0.2080^*^ | 7.41 | 0.00 |  | 0.2981 ± 0.2186^*^ | 9.64 | 0.00 |
| Right DLPFC-left hippocampus | 0.0531 ± 0.1870 | 1.99 | 0.05 |  | 0.0545 ± 0.1743^*^ | 2.21 | 0.03 |
| Right DLPFC-right hippocampus | 0.0535 ± 0.1575^*^ | 2.38 | 0.02 |  | 0.0332 ± 0.1783 | 1.32 | 0.19 |
| Left MPFC-left ACC | 0.0455 ± 0.2294 | 1.39 | 0.17 |  | −0.0221 ± 0.2118 | −0.74 | 0.46 |
| Left MPFC-right ACC | 0.0257 ± 0.2497 | 0.72 | 0.48 |  | −0.0511 ± 0.2471 | −1.46 | 0.15 |
| Left MPFC-left DLPFC | 0.1033 ± 0.1810^*^ | 3.99 | 0.00 |  | 0.1012 ± 0.1987^*^ | 3.60 | 0.00 |
| Left MPFC-right DLPFC | −0.0040 ± 0.1963 | -0.14 | 0.89 |  | −0.0318 ± 0.2056 | −1.09 | 0.28 |
| Left MPFC-right MPFC | 0.0687 ± 0.2102^*^ | 2.29 | 0.03 |  | 0.0385 ± 0.2139 | 1.27 | 0.21 |
| Left MPFC-left hippocampus | 0.0611 ± 0.1920^*^ | 2.23 | 0.03 |  | −0.0598 ± 0.1663^*^ | −2.54 | 0.01 |
| Left MPFC-right hippocampus | −0.0301 ± 0.2014 | −1.05 | 0.30 |  | −0.0514 ± 0.1499^*^ | −2.43 | 0.02 |
| Right MPFC-left ACC | 0.0130 ± 0.2290 | 0.40 | 0.69 |  | 0.0861 ± 0.2633^*^ | 2.31 | 0.03 |
| Right MPFC-right ACC | 0.0613 ± 0.2475 | 1.73 | 0.09 |  | 0.1037 ± 0.2857^*^ | 2.57 | 0.01 |
| Right MPFC-left DLPFC | 0.1146 ± 0.1663^*^ | 4.82 | 0.00 |  | 0.1136 ± 0.1946^*^ | 4.13 | 0.00 |
| Right MPFC-right DLPFC | 0.1751 ± 0.1557^*^ | 7.87 | 0.00 |  | 0.1965 ± 0.1744^*^ | 7.97 | 0.00 |
| Right MPFC-left MPFC | 0.1503 ± 0.1764^*^ | 5.97 | 0.00 |  | 0.1969 ± 0.2051^*^ | 6.79 | 0.00 |
| Right MPFC-left hippocampus | 0.0425 ± 0.1591 | 1.87 | 0.07 |  | −0.0099 ± 0.1367 | −0.51 | 0.61 |
| Right MPFC-right hippocampus | 0.0217 ± 0.1825 | 0.83 | 0.41 |  | −0.0071 ± 0.1460 | −0.34 | 0.73 |
| Left hippocampus-left ACC | 0.0553 ± 0.2922 | 1.33 | 0.19 |  | 0.0695 ± 0.2859 | 1.72 | 0.09 |
| Left hippocampus-right ACC | 0.0986 ± 0.3714 | 1.86 | 0.07 |  | 0.1038 ± 0.3491^*^ | 2.10 | 0.04 |
| Left hippocampus-left DLPFC | 0.0305 ± 0.2759 | 0.77 | 0.44 |  | 0.1099 ± 0.3088^*^ | 2.52 | 0.01 |
| Left hippocampus-right DLPFC | 0.0393 ± 0.2713 | 1.01 | 0.32 |  | 0.1012 ± 0.3161^*^ | 2.26 | 0.03 |
| Left hippocampus-left MPFC | 0.0620 ± 0.2660 | 1.63 | 0.11 |  | 0.0825 ± 0.2795^*^ | 2.09 | 0.04 |
| Left hippocampus-right MPFC | 0.0300 ± 0.2834 | 0.74 | 0.46 |  | 0.0961 ± 0.3044^*^ | 2.23 | 0.03 |
| Left hippocampus-right hippocampus | 0.1672 ± 0.3269^*^ | 3.58 | 0.00 |  | 0.2188 ± 0.3074^*^ | 5.03 | 0.00 |
| Right hippocampus-left ACC | 0.0934 ± 0.3193^*^ | 2.05 | 0.05 |  | 0.0823 ± 0.2694^*^ | 2.16 | 0.04 |
| Right hippocampus-right ACC | 0.0923 ± 0.3510 | 1.84 | 0.07 |  | 0.1485 ± 0.3468^*^ | 3.03 | 0.00 |
| Right hippocampus-left DLPFC | 0.0461 ± 0.3041 | 1.06 | 0.29 |  | 0.0844 ± 0.3066 | 1.95 | 0.06 |
| Right hippocampus-right DLPFC | 0.0693 ± 0.2612 | 1.86 | 0.07 |  | 0.1085 ± 0.3097^*^ | 2.48 | 0.02 |
| Right hippocampus-left MPFC | 0.0735 ± 0.2661 | 1.93 | 0.06 |  | 0.0675 ± 0.2667 | 1.79 | 0.08 |
| Right hippocampus-right MPFC | 0.0457 ± 0.2851 | 1.12 | 0.27 |  | 0.0951 ± 0.2867^*^ | 2.34 | 0.02 |
| Right hippocampus-left hippocampus | 0.2198 ± 0.2791^*^ | 5.51 | 0.00 |  | 0.2513 ± 0.2341^*^ | 7.59 | 0.00 |

^*^Significant effective connectivity at the group level (*P* < 0.05, Bonferroni corrected). ACC, anterior cingulate cortex; DLPFC, dorsolateral prefrontal cortex; MPFC, medial prefrontal cortex.
